# A predictive model for respiratory syncytial virus (RSV) hospitalisation of premature infants born at 33–35 weeks of gestational age, based on data from the Spanish FLIP study

**DOI:** 10.1186/1465-9921-9-78

**Published:** 2008-12-08

**Authors:** Eric AF Simões, Xavier Carbonell-Estrany, John R Fullarton, Johannes G Liese, Jose Figueras-Aloy, Gunther Doering, Juana Guzman, European RSV Risk Factor Study Group

**Affiliations:** 1 Professor of Pediatrics, Department of Pediatrics, Section of Infectious Diseases, The University of Colorado School of Medicine and The Children's Hospital, Denver, Colorado, USA; 2 Neonatology Service, Hospital Clínic, Institut Clínic de Ginecologia Obstetricia i Neonatologia, Agrupació Sanitaria Hospital Clínic-Hospital SJ Deu, Universitat de Barcelona, Barcelona,Spain; 3 Analyst, Strategen Limited, Basingstoke, Hampshire, UK; 4 Dr. von Hauner Children's Hospital, Ludwig-Maximilians-University, Munich, Germany; 5 Neonatology Service, Hospital Clínic, Institut Clínic de Ginecologia Obstetricia i Neonatologia, Agrupació Sanitaria Hospital Clínic-Hospital SJ Deu, Universitat de Barcelona, Barcelona, Spain; 6 Munich University of Technology, Department of Pediatrics, Munich, Germany; 7 Hospital Reina Sofía, Córdoba, Spain

## Abstract

**Background:**

The aim of this study, conducted in Europe, was to develop a validated risk factor based model to predict RSV-related hospitalisation in premature infants born 33–35 weeks' gestational age (GA).

**Methods:**

The predictive model was developed using risk factors captured in the Spanish FLIP dataset, a case-control study of 183 premature infants born between 33–35 weeks' GA who were hospitalised with RSV, and 371 age-matched controls. The model was validated internally by 100-fold bootstrapping. Discriminant function analysis was used to analyse combinations of risk factors to predict RSV hospitalisation. Successive models were chosen that had the highest probability for discriminating between hospitalised and non-hospitalised infants. Receiver operating characteristic (ROC) curves were plotted.

**Results:**

An initial 15 variable model was produced with a discriminant function of 72% and an area under the ROC curve of 0.795. A step-wise reduction exercise, alongside recalculations of some variables, produced a final model consisting of 7 variables: birth ± 10 weeks of start of season, birth weight, breast feeding for ≤ 2 months, siblings ≥ 2 years, family members with atopy, family members with wheeze, and gender. The discrimination of this model was 71% and the area under the ROC curve was 0.791. At the 0.75 sensitivity intercept, the false positive fraction was 0.33. The 100-fold bootstrapping resulted in a mean discriminant function of 72% (standard deviation: 2.18) and a median area under the ROC curve of 0.785 (range: 0.768–0.790), indicating a good internal validation. The calculated NNT for intervention to treat all at risk patients with a 75% level of protection was 11.7 (95% confidence interval: 9.5–13.6).

**Conclusion:**

A robust model based on seven risk factors was developed, which is able to predict which premature infants born between 33–35 weeks' GA are at highest risk of hospitalisation from RSV. The model could be used to optimise prophylaxis with palivizumab across Europe.

## Background

Respiratory syncytial virus (RSV) causes a severe lower respiratory tract disease that results in substantial morbidity in premature infants [1,2]. Infants born up to 35 weeks' gestational age (wGA) lack the necessary pulmonary and immunologic development and function essential to combating infection [3-5]. It is estimated that 1–3% of previously healthy infants are hospitalised because of RSV infection [6], whereas the RSV-hospitalisation rate ranges between 3.75% and 9.8% for infants born between 33–35 wGA [1,7,8]. Studies suggest that infants born between 33–35 wGA are at risk of developing severe RSV infection that can result in morbidity and health care resource utilisation similar to infants born ≤ 32 wGA [9,10]. Additionally, RSV-related hospitalisation in 32–35 wGA infants causes significant morbidity and healthcare utilisation in the subsequent years [11].

Palivizumab, a humanised monoclonal antibody, has been proven a safe and efficacious option to significantly reduce RSV disease in prematurely born infants up to and including 35 wGA [12-14]. Based on the findings of the pivotal Phase III trial (IMpact RSV Study) [12], palivizumab received European approval in 1999 for use in infants up to and including 35 wGA [15]. Despite the clinical evidence, only a few countries in Europe make passive immunoprophylaxis available to at-risk 33–35 wGA infants, as reflected in current national guideline and reimbursement policies [16-18]. Passive immunoprophylaxis for all infants born at 33–35 wGA is not financially viable. However, based on risk profile and a higher rate of RSV-related hospitalisation, a certain proportion of these infants may be legitimate candidates for prophylaxis.

A comprehensive review of the literature revealed environmental and demographic risk factors that predispose infants to developing severe RSV leading to hospitalisation [19]. Subsequent prospective studies in Spain [9], Canada [7], and Germany [20] examined those risk factors in infants born 33–35 wGA. The risk factors identified include: chronological age, number of siblings/contacts, history of atopy, absence/duration of breast feeding, postnatal cigarette smoke exposure, male sex, and day care attendance [7,9,20]. Despite these data, no predictive tool that can identify infants most at risk of RSV-hospitalisation has been developed. We have developed an objective, evidence-based model to assist clinicians to predict the likelihood of RSV hospitalisation in European infants born 33–35 wGA. Such a model would facilitate the effective and responsible application of passive immunoprophylaxis in this population.

## Methods

### Population used for modelling

The predictive model was derived from the Spanish FLIP dataset [9], a prospective, case-control study, which aimed to identify those risk factors most likely to lead to the development of RSV-related hospitalisation among premature infants born at 33–35 wGA. The dataset comprises 186 cases and 371 age-matched controls recruited from 50 centres across Spain during the 2002/2003 RSV season (Oct. 2002-Apr. 2003). Criteria for inclusion as a case included: GA between 33–35 weeks, discharge during the RSV season (or age ≤ 6 months at the start of the RSV season), and proven RSV-related hospitalisation. Controls were selected from premature infants born or discharged from the same hospital, during the same time period, and within the same GA limits as cases, but who had not been previously hospitalised for any acute respiratory illness during the RSV season. Additionally, although not a criterion for study exclusion, no infant had chronic lung disease.

### Statistical methodology

Discriminant function analysis [21] was used to build the predictive model. Univariate analyses included the Student's *t *test, the χ^2 ^test, the Mann-Whitney's *U *test, and the calculation of odds ratios (with 95% confidence intervals). The model was internally validated using bootstrapping methods [22]. All data were analysed by SPSS software (version 10) [23]. Records with missing values for one or more of the predictor variables were excluded from the analyses.

#### Development of a model to predict RSV-related hospitalisation of infants 33–35 wGA

All the available risk factors collected in the FLIP study were included in the discriminant analysis. The discriminant analysis established how well the presence or absence of certain risk factors was able to separate infants in the hospitalised group from those in the non-hospitalised group (generating a discriminant function).

Following the development of an initial model, backward selection was used to remove the variables that contributed least to the discriminant function. The elimination of a variable from the analysis was based on a comparison of the discriminant power of the function derived with and without the variable. At each stage, the functions for each reanalysis were compared to identify the most discriminatory.

Receiver operator characteristic (ROC) curves were constructed by plotting the sensitivity against 1-the specificity. The area under the curve was calculated for each ROC plot, with areas closer to 1 representing better predictive accuracy. To explore diagnostic accuracy, positive predictive values (PPV), negative predictive values (NPV), and likelihood ratios were generated [24,25]. Additionally, example numbers needed to treat (NNT) were calculated.

#### Validation of the predictive model

The FLIP dataset was subject to 100-fold bootstrapping validation [22]. For each of the 100 samples, coefficients for each predictor variable were calculated. The 100 coefficient sets were then used to derive predictor functions on 100 replicates of the original data. The correct prediction of RSV-related hospitalisation was calculated and ROC curves were plotted for each of the 100 outputs. The distribution of correct prediction rates and areas under the ROC curve were then assessed. To test for normality in the distribution of correct prediction rates and areas under the ROC curve, the Kolmogorov-Smirnov test was used [26]. The results were also tested for skewness.

#### Test of the predictive model against an external dataset

Despite extensive investigation, there were no suitable European datasets available against which the model could be fully externally validated. Therefore, to gain a measure of the applicability of the model to other European populations, the model was tested against data from the Munich RSV study [8]. The Munich RSV study, a population based cohort study, examined the incidence and risk factors for RSV-related hospitalisation of premature infants born ≤ 35 wGA. Questionnaires were sent to all parents of infants discharged from primary neonatal care to determine the event of rehospitalisation for acute respiratory infections. A total of 717 infants were studied, 375 of whom were born between 33–35 wGA and were used in the validation. There were 37 RSV-related hospitalisations (5.2%) overall and 20 amongst the 375 preterms of 33–35 wGA (5.3%). Of the 20 RSV-related hospitalisations, six had a confirmed diagnosis of RSV, with the remaining 14 cases being classified as having a clinical suspicion of RSV, although two had a negative RSV test on one occasion. The two infants with a negative RSV test were excluded from the analysis.

The predictive function derived from the FLIP dataset was tested in two ways against data from the Munich RSV study. Firstly, the predictive variables identified from the FLIP dataset were used to generate a discriminant function from the data of the Munich RSV study itself. Secondly, the non-normalised coefficients (derived from unadjusted variable data) generated from the FLIP dataset were applied to the Munich data.

Prior to testing, the final model had to be adjusted to account for differences in the data captured within the FLIP study and that which were captured within the Munich RSV study. The variable 'number of family members with wheeze' had to be removed, as this was not available in the Munich dataset, the variable 'breast fed for ≤ 2 months or not' had to be modified to 'breast fed Yes/No', and the variable 'number of family members with atopy' had to be changed to a categorical 'family member with atopy Yes/No'.

#### Test of the predictive model against the Spanish Guidelines recommendations for prophylaxis of 32–35 wGA infants

To put the clinical usefulness of the model into perspective, its predictive ability was compared to that based on the Spanish Neonatal Society Guidelines [16] recommendations for prophylaxis of infants born 32–35 wGA. The Spanish Guidelines [16] recommend that premature infants born 32–35 wGA who are ≤ 6 months old when the RSV season starts and have two risk factors (less than 10 weeks when RSV season starts, tobacco smoke at home, day care assistance, no breast feeding, family history of wheezing, school age siblings, and crowded homes [≥ 4 residents and/or visitors at home, excluding school age siblings and the subject him/herself]) receive prophylaxis with palivizumab. Using these criteria, a discriminant function was generated from the FLIP dataset, a ROC curve plotted, and diagnostic accuracy tested. The results from this analysis were then compared to the results for the model.

## Results

### Development of the predictive model

The 15 variables in the FLIP study are compared in the hospitalised and non-hospitalised infants in Table 1. In a univariate analysis of the FLIP data, hospitalised infants were significantly more likely to be born within 10 weeks of the start of the RSV season, be heavier at birth, have more family members with atopy or who wheezed, had more carers at home, had mothers who smoked during pregnancy, had more siblings ≥ 2 years of age, and were breast fed for ≤ 2 months or not at all.

**Table 1:** A comparison of the risk factors for RSV hospitalised and non hospitalised infants in the FLIP and Munich studies^†^

	**FLIP **[9]				**Munich **[8]			
	
	Hospitalised (n = 186)	Non-hospitalised (n = 367)	Odds Ratio (CI 95%)	P-value*	Hospitalised (n = 20)	Non-hospitalised (n = 357)	Odds Ratio (CI 95%)	P-value*
*Birth ± 10 weeks of start of season*	*136 (73.1%)*	*145 (39.5%)*	*4.16 (2.78–6.23)*	**<*0.0001***	*12 (60.0%)*	*148 (41.5%)*	*2.12 (0.77–6.12)*	*0.1101*

*Birth weight, kg*^*a*^	*2.20 (0.38)*	*2.12 (0.42)*	*-*	***0.0419***	*2.14 (0.38)*	*2.11 (0.39)*	*-*	*0.7526*

*Breast fed ≤ 2 months or not*^*§*^	*146 (78.5%)*	*206 (56.1%)*	*2.85 (1.87–4.40)*	**<*0.0001***	*18 (90.0%)*	*286 (80.1%)*	*2.23 (0.51–20.3)*	*0.3887*

*Number of siblings ≥ 2 years*	*1 (0–1)*	*0 (0–1)*	*-*	**<*0.0001***	*1 (0–2)*	*0 (0–1)*	*-*	***0.0172***

*Number of family with atopy*^*§*^	*0 (0-0)*	*0 (0-0)*	*-*	***0.0117***	*12 (60.0%)*	*175 (49.0%)*	*1.56 (0.57–4.51)*	*0.3671*

*Male gender*	*117 (62.9%)*	*199 (54.2%)*	*1.43 (0.98–2.09)*	*0.0513*	*18 (90.0%)*	*177 (49.6%)*	*9.15 (2.13–82.14)*	***0.0003***

*Number of family with wheeze*	*0 (0–1)*	*0 (0-0)*	*-*	***0.0004***	*-*	*-*	*-*	*-*

Gestational age								
33 weeks	49 (26.3%)	77 (21.0%)	1.34 (0.87–2.07)	0.1554	4 (20.0%)	119 (33.3%)	0.50 (0.12–1.60)	0.3265
34 weeks	60 (32.3%)	139 (37.9%)	0.78 (0.53–1.15)	0.1935	11 (55.0%)	172 (48.2%)	1.31 (0.48–3.68)	0.648
35 weeks	77 (41.4%)	151 (41.1%)	1.01 (0.69–1.47)	0.9544	5 (25.0%)	66 (18.5%)	1.47 (0.40–4.44)	0.5544

Number of regular carers	2 (1–2)	2 (1–2)	-	**0.0377**	-	-	-	-

Furred pets at home	46 (24.7%)	68 (18.5%)	1.44 (0.92–2.25)	0.0885	-	-	-	-

Educational level of parents								
No school	7 (3.8%)	4 (1.1%)	3.54 (0.89–16.71)	0.0711	-	-	-	-
Primary	53 (28.5%)	84 (22.9%)	1.34 (0.88–2.04)	0.1491	-	-	-	-
High school	78 (41.9%)	156 (42.5%)	0.98 (0.67–1.42)	0.8978	-	-	-	-
University	48 (25.8%)	123 (33.5%)	0.69 (0.45–1.04)	0.0639	-	-	-	-

Number of births in delivery	1 (1–2)	1 (1–2)	-	0.531	1 (1-1)	1 (1–2)	-	0.1675

Smoking during pregnancy^*b*^	56 (30.3%)	79 (21.5%)	1.58 (1.03–2.40)	**0.0241**	-	-	-	-

Number of smokers around infant^*c*^	1 (0–2)	1 (0–2)	-	0.062	0 (0–1)	0 (0–1)	-	0.9479

Number of family with asthma	0 (0-0)	0 (0-0)	-	0.1114	-	-	-	-

The 8 variables used in the final model are shown in italics. All variables were used in the initial 15 variable model

† Mean (standard deviation), median (P25-P75), number (%)

* Student's *t *test, Mann-Whitney *U *test, χ^2 ^test

§Recorded as breast fed yes/no and atopy yes/no for Munich

a 2 missing values for FLIP, 5 missing values for Munich

b 1 missing value for FLIP

c 2 missing values for Munich

**Figure 1:**
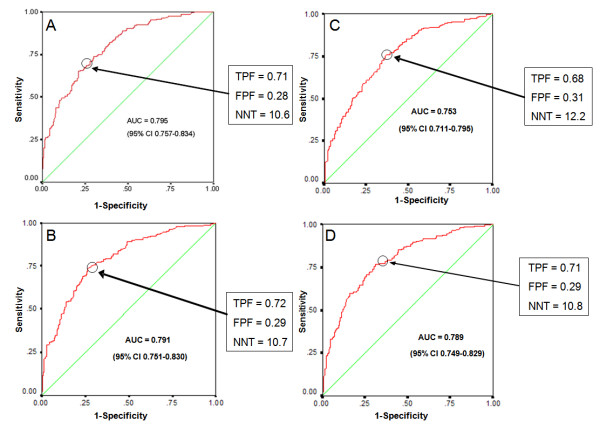
**Receiver operating characteristic (ROC) curves for 15 variable model (A), final 7 variable model (B), 6 variable model for Munich test (C), and 6 variable model with sex removed (D)**. The number needed to treat (NNT) at the point of maximum sensitivity/specificity is based on a hospitalisation rate of 5% and a treatment efficacy of 80%. Each point on the ROC curve represents a case being either a true positive or a false positive, based on their discriminant score. CI = confidence interval; TPF = true positive fraction; FPF = false positive fraction.

The initial analysis of the FLIP dataset produced a function based on 15 risk factors, which could discriminate significantly between hospitalised and non-hospitalised infants. This function could correctly classify whether a child was hospitalised or not in 72% of cases (table 2). Importantly, the correct classification of hospitalised infants was 71%. The area under the ROC curve was 0.795 (Figure 1A).

**Table 2:** Analyses of the predictive accuracy of the various models

	**True Positive**	**False Positive**	**False Negative**	**True Negative**	**Sensitivity**	**Specificity**	**PPV** **%**	**NPV** **%**	**LR**	**Diagnostic Accuracy %**
	
**FLIP 15 variable model^§^**	130	102	53	265	0.71	0.72	56	83	2.56	72
**FLIP Final 7 variable model^¤^**	139	113	45	254	0.76	0.69	55	85	2.45	71

**Munich 6 variable model^†^**	14	106	4	247	0.78	0.70	12	98	2.59	70

^§ ^Records for 550 infants were included within the analysis. Seven records were dropped from the analysis due to missing data for one or more of the predictor variables

^¤ ^Records for 549 infants were included within the analysis. 8 records were dropped from the analysis due to missing data for one or more of the predictor variables

^† ^Records for 370 infants were included within the analysis. Three records were dropped from the analysis due to missing data for one or more of the predictor variables. Two records for hospitalised cases were removed from the analysis, as they each had one negative RSV test

PPV = positive predictive value

NPV = negative predictive value

LR = likelihood ratio of a positive test; for information about likelihood ratios see reference 25

Standardised canonical discriminant function coefficients for the FLIP final 7 variable model: birth ± 10 weeks of start of season = 0.678, birth weight, kg = 0.184, breast fed ≤ 2 months or not = 0.511, number of siblings ≥ 2 years = 0.489, number of family with atopy = 0.151, female sex = -0.113, number of family with wheeze = 0.125

The variable reduction exercise resulted in a final model of seven variables (table 1 in italics), with an area under the ROC curve very similar to that of the 15 variable model (Figure 1B). Discrimination also remained similar at 71%, with 76% of hospitalisations classified correctly (table 2). At the 0.75 sensitivity intercept, the specificity was 0.67, with the false positive fraction (FPF) being 0.33. The NNT to prevent hospitalisation of 75% of at risk infants was calculated to be 11.7, assuming a 5% hospitalisation rate (consensus of European RSV Risk Factor Study Group based on a review of the available data [1,7,8]) and 80% [12] treatment efficacy (table 3). At the point of maximum sensitivity/specificity the NNT was 10.7, again assuming a 5% hospitalisation rate and 80% treatment efficacy (Figure 1). The likelihood ratio for this model was 2.45 and the PPV and NPV were 55% and 85%, respectively.

**Table 3:** Final seven variable model number needed to treat analyses*

**ROC AUC plus confidence limits**	**True Positive Fraction**	**True positives treated**	**False Positive Fraction**	**False positives treated**	**NNT**	**NNT** **(80% efficacy)**
0.791 (mid point)	0.75	75	0.33	627	9.4	11.7

0.751 (lower limit)	0.75	75	0.39	741	10.9	13.6

0.830 (upper limit)	0.75	75	0.26	494	7.6	9.5

*Number needed to treat (NNT) to prevent hospitalisation of 75% of at risk infants, assuming a 5% hospitalisation rate and 80% treatment efficacy (n = 2,000)

### Contribution of individual variables

A variable reduction exercise on the 7 variable model showed that, although some variables were more important than others, removing any variable produces a decrease in discrimination and/or area under the ROC curve. For example, removing 'sex' reduced the area under the ROC curve to 0.789 (Figure 1D). On this basis, no clear case could be made for removing any of the constituent seven variables. Thus, the final seven variable model includes: birth within 10 weeks of the start of season, birth weight, breast fed for ≤ 2 months or not, number of siblings ≥ 2 years, number of family members with atopy, male sex, and number of family members with wheeze.

### Validation

The bootstrapping analysis resulted in a tight symmetrical distribution of results for the 100 calculations of percentage correctly predicted and area under the ROC curve (table 4). The mean percentage of cases predicted correctly was 72% (standard deviation [SD]: 2.18) and the median area under the ROC curve was 0.785 (range 0.768–0.790). The Kolmogorov-Smirnov test indicated that the distribution of results for the correct prediction of outcomes (asymptotic significance: P = 0.910) and for the ROC curves (asymptotic significance: P = 0.101) is assumed to be normal for the purposes of calculation. Calculation of the skewness statistic found no indication of skewness in the distribution of results for the correct prediction of outcomes (0.19, two standard errors of skewness [SES]: 0.48), but did find significant skewness in the area under the ROC curve results (-1.20, 2 × SES: 0.48). However, a Q-Q plot for the areas under the ROC curve suggests that the deviation from normality was symmetrical (figure not shown). In summary, this means that two SDs for the correct prediction of hospitalisation (2 × 2.18 = 4.36) can be take as the 95% CI for the results i.e. 72% ± 4.36.

**Table 4:** 100-fold bootstrap statistics on the FLIP dataset

	**Percentages correctly predicted**	**Areas under ROC curves (AUC)**
**Mean**	72.00	0.784

**Median**	72.20	0.785

**Standard deviation**	2.18	0.004

**Minimum**	66.20	0.768

**Maximum**	77.40	0.790

**Kolmogorov-Smirnov Z**	0.56 (P = 0.910^†^)	1.22 (P = 0.101^†^)

**Skewness statistic**	0.19 (0.48^§^)	-1.20 (0.48^§^)

n = valid: 100, missing: 0

^† ^Asymptotic significance (2-tailed)

^§ ^2 standard error of skewness

### External Test

The Munich dataset did not include numbers of family members with wheeze, so coefficients obtained for the remaining six variables of the seven variable model were used. The recalculated six variable model was somewhat weaker than the seven variable model defined earlier. However, its power, derived by running the model on the FLIP data, was adequate for running the validation tests (correct classification: 68%; area under ROC curve 0.753 (Figure 1C).

When we used the six variables identified in the FLIP study to derive coefficients from the Munich dataset, the function derived solely from the Munich data was comparable to that obtained with the FLIP dataset (correct classification: 70% [table 2]; area under ROC curve 0.812, 95% CI 0.737–0.887). Applying the FLIP derived coefficients (from the seven variable model) to the Munich data produced a function that could correctly classify 64% of cases, with an area under the ROC curve of 0.677 (95% CI 0.551–0.804).

### Spanish Guidelines Test

The discriminant function based on the guidelines recommendations could correctly classify 38% of cases – which is no better then chance – and had an area under the ROC curve of 0.520 (95% CI 0.468–0.573). The PPV was 36%, the NPV 100%, and the likelihood ratio 1.04. (It is worth remembering that a completely non-discriminatory test that selects all patients for treatment except one, would have a NPV of 100% if this patient were truly negative.) Based on a 5% hospitalisation rate and 80% efficacy, the NNT to prevent hospitalisation of 75% of at risk infants was calculated to be 24.7.

## Discussion

We have developed and validated a robust European predictive model to identify the risk of RSV-related hospitalisation in infants born between 33–35 wGA. The FLIP 7-variable model correctly classifies over 70% of cases, which, to put into context, compares to a figure of 38% when using the Spanish Guidelines [16] for prophylaxis. The predictive ability of the model was confirmed through validation. The tight symmetrical distributions for both the correct predictions of hospitalisation and area under the ROC curve results and the mostly convex nature of the ROC curve demonstrate that the model is not skewed by 'outliers' in the FLIP dataset and is, therefore, highly reproducible however the data may be sampled. This lends a high degree of confidence to the model derived from the FLIP dataset.

The seven variables used in the final model were 'birth within 10 weeks of the start of season', 'birth weight', 'breast fed for ≤ 2 months or not', 'number of siblings ≥ 2 years', 'number of family members with atopy', 'male sex', and 'number of family members with wheeze'. All of these variables have been documented as risk factors for RSV-related hospitalisation [7,19,20,27]. Indeed, a critical evaluation of the literature concluded that 'male sex' and 'crowding/siblings' were significant risk factors for severe RSV lower respiratory tract infection [19]. However, the same review also reported that a lack of breast feeding did not appear to increase the risk of severe RSV lower respiratory tract infection or RSV-related hospitalisation [19]. A recently published nested case-control study supports that familial atopy and wheezing are strong determinants of RSV-related hospitalisation [27].

The strength and utility of the FLIP 7-variable model was highlighted by an examination of NNT. Assuming a 5% hospitalisation rate and 80% treatment efficacy, the calculated NNT to prevent hospitalisation of 75% of at-risk patients was 11 (range 10–14). A NNT of 11 is better than half the result if infants are prophylaxed based on the Spanish Guidelines recommendations [16] (25) and is considerably lower than the 17 obtained from using the raw numbers of the IMpact-RSV trial [12].

Although various analytical approaches were considered, it was decided to develop the model using discriminant function analysis. This approach produced similar results to logistic regression, but was arguably more applicable in the manipulation involved in validation, such as handling missing values and continuous data. Further, models derived from discriminant function analysis can benefit from the inclusion of variables that are not independently significant, but which contribute to the overall predictive ability of the model. Indeed, the discriminatory power of such models is always greater than that afforded by the simple sum of its component parts. To exemplify this, one of the seven variables in the final model was not independently significant (male sex), but is a well known risk factor [19]. The model also has good flexibility, as the sensitivity and specificity along the ROC curve can easily be varied such that different cut-off points can be selected and NNTs calculated according to the needs of the individual European country.

As is the case whenever developing such a model, limitations were imposed by what and how data were captured within the base dataset. Although the FLIP study [9] contained a great deal of information on risk factors and hospitalisation rates for children born between 33–35 weeks' GA, it was limited by being a case-control study. Since RSV infection had to be proven and these were likely to have been the most severe cases, this might have lead to selection and, therefore, bias in the dataset. Further, allowance had to be made for the variability in admission criteria for the various hospitals across Spain. Finally, since day care attendance is not commonly practised in Spain, there were limited data on this variable and it was not included in the final analyses.

External validation of the model presented a challenge as there were no suitable databases in Europe that were available for such a purpose. As a surrogate, the model was tested against data from the Munich RSV study. Allowances have to be made for the differences in how the study was conducted and what data were captured compared with FLIP. For example, no data were captured on wheeze in the Munich study. Perhaps most significantly, data were available for only 20 hospitalised infants within the Munich study. Further, only six of the hospitalised infants had a confirmed diagnosis of RSV, as testing is not routine in Germany. Taking these differences into consideration, the test can be considered a worse case scenario, as it would be not be expected for the model to validate particularly well against the Munich data. However, despite these significant limitations, the FLIP model tested very well against the Munich data. Nevertheless, rigorous external validations of the model are planned when suitable prospective data become available within Europe over the next couple of years.

A recently published Dutch model [28], which estimated the monthly risk of hospitalisation, reported that gender, GA, birth weight, presence of bronchopulmonary dysplasia, age, and seasonal monthly RSV pattern were significant predictors and could potentially be used to discriminate between high and low risk children. The Dutch model included only risk factors that were reported as independently significant in the published literature. In comparison, all risk factors available within the FLIP dataset were included within our modelling, regardless of their individual significance. In addition, the Dutch model does not specifically address the group we are trying to predict RSV-related hospitalisation within, namely, those infants born 33–35 wGA without CLD. Finally, the Dutch model imputed missing values, whereas in the development of the FLIP model, patients with incomplete records were excluded from the analyses. Several other studies have proposed using identified risk factors to predict RSV hospitalisation in premature infants [7,20,29]; however, as far as the authors are aware, no other models or scoring systems have been formally published.

Importantly, although the significance of the individual risk factors may vary between countries, the validation and testing process indicates that the model may be applicable for widespread use across Europe. Moreover, the model appears flexible yet robust enough that, if necessary, individual variable parameters can be modified to suit the needs of individual countries. Further, although the model is suitable for adoption as it stands, countries could use their own data, either existing or prospectively collected, to refine a predictive tool. When considering intervention levels within a predictive tool, variation in hospitalisation rates for RSV across different countries would not affect the performance of the model in terms of prediction, as this is not factored into the analysis.

The model could be realised as a working tool in a variety of formats to optimise its applicability to an individual country, or, indeed, an individual unit. Formats could potentially include a bespoke software application, a website, a simple spreadsheet, or even a paper-based form or nomogram. The big advantage of a software application or website is that either could prospectively capture risk factors and outcomes data, which could be used to further refine and validate the model and justify its continuing use. The tool itself would be used in daily practice to predict the risk of RSV-related hospitalisation for individual infants. Chronic conditions such as CLD, congenital heart disease, and severe neurological diseases may further increase the risk of RSV-related hospitalisation, and, therefore, should always be taken into consideration when using the tool.

## Conclusion

By using data from the Spanish FLIP study [9] and carrying out validation, we have produced an evidenced-based model which is applicable for adaptation and use in different countries across Europe. The model has the potential to improve standards of care by better identifying high risk infants and, thus, optimising prophylaxis. It may also be used to inform guidance and to help clarify the justification of funding and reimbursement for palivizumab within health services. Finally, this study has led to a better understanding of the risk factors and their interrelationships for infants born between 33–35 weeks' GA.

## Competing interests

JF has received fees from Abbott Laboratories for work on various projects. XCE, ES, GD and JL have acted as expert advisors and speakers for Abbott Laboratories and have received honoraria in this regard.

## Authors' contributions

XCE, ES, JL, and JF contributed to the concept and design of the model. JF carried out the statistical modelling with input from XCE, ES, and JL. XCE, ES, JL undertook the clinical interpretation of the data. All authors contributed to the manuscript.
